# Ten Years of Research on Fucoidan and Cancer: Focus on Its Antiangiogenic and Antimetastatic Effects

**DOI:** 10.3390/md21050307

**Published:** 2023-05-18

**Authors:** Eleonora Turrini, Francesca Maffei, Carmela Fimognari

**Affiliations:** Department for Life Quality Studies, University of Bologna—C.so d’Augusto, 237, 47921 Rimini, Italy; eleonora.turrini@unibo.it (E.T.); francesca.maffei@unibo.it (F.M.)

**Keywords:** marine polysaccharides, fucoidans, anticancer drugs, antiangiogenic activity, antimetastatic activity

## Abstract

Angiogenesis and metastasis represent two challenging targets to combat cancer development in the later stages of its progression. Numerous studies have indicated the important role of natural products in blocking tumor angiogenesis signaling pathways in several advanced tumors. In recent years, the marine polysaccharides fucoidans emerged as promising anticancer compounds showing potent antitumor activity in both in vitro and in vivo models of different types of cancers. The objective of this review is to focus on the antiangiogenic and antimetastatic activities of fucoidans with special emphasis on preclinical studies. Independently from their source, fucoidans inhibit several angiogenic regulators, primarily vascular endothelial growth factor (VEGF). A glance towards fucoidans’ ongoing clinical trials and pharmacokinetic profile is provided to present the main challenges that still need to be addressed for their bench-to-bedside translation.

## 1. Introduction

Cancer still represents one of the main causes of death worldwide, accounting for more than 9.6 million deaths in 2018 [1]. The design, identification, and evaluation of novel anticancer agents continue to represent an active field of research, where cancer angiogenesis and metastatization appear to be two of the most challenging therapeutic targets [2]. Angiogenesis is a complex physiological process leading to new vessel formation from preexisting vasculature: it is a key step in tumor progression and recurrence and is known as a hallmark of solid tumors [3]. The angiogenetic process encompasses the increase in vasopermeability, migration of endothelial cells via extracellular matrix (ECM) degradation, differentiation, and the formation and maturation of new vessels. The formation and development of a vascular network are crucial for sustaining the adequate supply of oxygen and nutrients along with the removal of waste from cancer cells, allowing the grow and unrestricted proliferation of tumors. In addition, direct contact with the vascular network allows the tumor to metastasize and spread beyond the primary site. However, cancer vessels are characterized by an abnormal configuration of their surrounding cells, leaks, and various malfunctions compared with normal vasculature. These differences may be useful in the design of new anticancer drugs [4].

Most of the approved antiangiogenic drugs are targeted therapies directed towards the vascular endothelial growth factor (VEGF), its receptors, or specific molecules involved in angiogenesis. Antiangiogenic drugs can also be used as chemosensitizers of anticancer strategies such as chemotherapy, target therapies, and immune checkpoint inhibitors in several advanced tumors [4].

Numerous studies have indicated the important role of natural products in the oncological area [5,6]. In particular, several natural compounds have been found to target tumor-angiogenesis-associated cytokines, and apoptotic signaling pathways, such as curcumin, artemisinin, epigallocatechin gallate, resveratrol, emodin, celastrol, thymoquinone, plocabulin, and tocotrienols, have all shown prominent anti-angiogenic effects in the preclinical models of tumor angiogenesis [7,8,9,10,11].

In recent years, marine algae polysaccharides emerged as promising anticancer compounds, exerting their activity via apoptosis induction, cell-cycle arrest, angiogenesis and metastasis suppression, immune response modulation, and regulation of gut microbiota [12].

**Figure 1:**
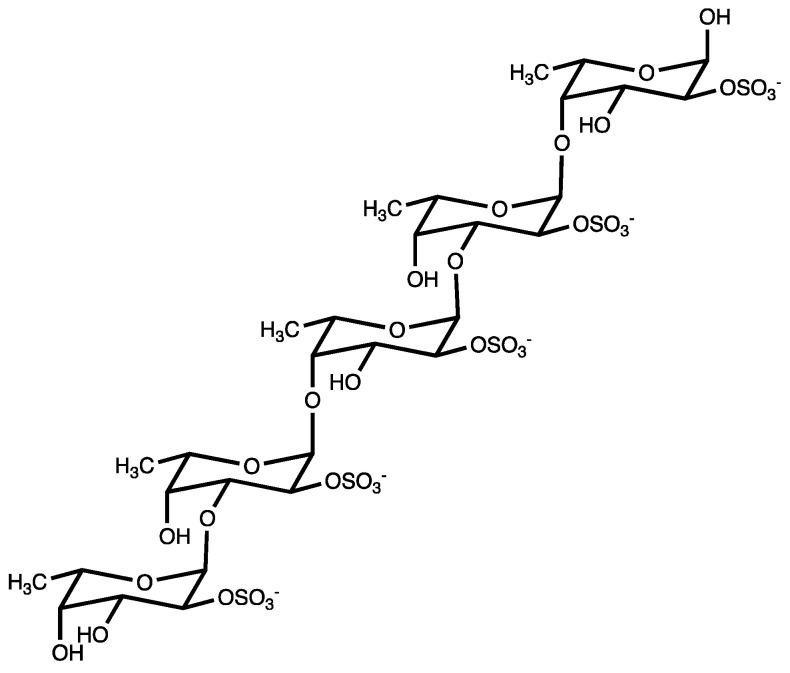
Fucoidan from brown seaweed.

“Fucoidans” are a class of polysulphated polysaccharides, mainly characterized by the presence of the monosaccharide L-fucose-4-sulfate (Figure 1). Structurally, fucoidan is a heparin-like molecule and, together with the substantial percentage of L-fucose and sulfated ester groups, is composed by small proportions of D-xylose, D-galactose, D-mannose, L-rhamnose, arabinose, glucose, acetyl groups, and glucuronic acid, depending on the source [13]. Fucoidans are extracted from the cell walls of different species of brown seaweeds such as *Fucus vesiculosus*, *Cladosiphon okamuranus*, *Sargassum polycystum*, *Laminaria japonica*, and *Undaria pinnatifida*. Their main function consists of sustaining cell membrane stability and protecting seaweeds against dehydration [14].

Fucoidans are extensively used in cosmetics and as food/dietary supplements, especially thanks to the low-toxic nature of those compounds [14,15]. Moreover, they are attracting increasing attention for their biological activities, such as anti-oxidant, antiviral, immunoregulatory, anti-coagulant, anti-thrombotic, anti-lipidemic, anti-diabetic, anti-tumor, anti-metastatic, and anti-angiogenic properties [13,16,17,18,19,20].

This review provides a comprehensive and critical analysis of the last 10 years of literature about the antiangiogenic and antimigratory activities of fucoidans explored in in vitro and in vivo experimental models. The recent clinical evidence on the benefit of fucoidans for cancer management is also discussed.

## 2. Antiangiogenic and Antimigratory Effects of Fucoidans

Fucoidans have demonstrated both antiangiogenic and antimigratory effects in different experimental models, independently from the source, purity, molecular weight (MW), chemical structure, and fucose or sugar content of the polysaccharide, as shown in Table 1. The studies are presented in the table based on the source of the fucoidan and in order of increasing complexity of the experimental model.

### 2.1. Antiangiogenic and Antimigratory Activity of Fucoidans In Vitro

Cancer cells and endothelial cells are two in vitro experimental systems that can give different and complementary information about the antiangiogenic and antimigratory potential of fucoidans. In particular, both can provide inside in the molecular mechanisms evoked by the treatment with fucoidans, such as the modulation of the expression or release of angiogenic targets. Moreover, they allow us to investigate the anti-invasive and anti-migratory potential of fucoidans using, for instance, the transwell assay. Endothelial cells can also provide information on new vessel formation or vascular density and, in co-culture with cancer cells, can mimic in vitro tumor microenvironments [44].

#### 2.1.1. Cancer Cell Lines

The process of angiogenesis is mainly triggered by hypoxia, as well as various other stimuli such as acidic pH, hypoglycemia, hypertension, mechanical stress, chronic inflammation, and oxidative stress, which can either initiate or accelerate angiogenesis [4]. Tissues sensitive to hypoxia release HIF-1α (hypoxia-inducible factor-1α), which binds the hypoxia response elements and activates the transcription of other pro-angiogenic factors. Increasing evidence supports the hypothesis that the PI3K (phosphatidylinositol-3 kinase)/Akt (protein kinase B)/mTOR (mammalian target of rapamycin) pathway acts as a master switch controlling HIF-1α synthesis. HIF-1α may activate the transcription of over 40 genes, including erythropoietin, glucose transporters, glycolytic enzymes, VEGFs (vascular endothelial growth factors) and other genes whose protein products increase oxygen delivery or facilitate metabolic adaptation to hypoxia, as well as promoting angiogenesis, tumor invasion and metastasis, and resistance to therapy [45].

The best-known angiogenesis regulator is the VEGF family including the proteins VEGFA, VEGFB, VEGFC, VEGFD, PGF (placental growth factor), VEGFE, and svVEGF (snake venom VEGF), which bind with different affinities to three tyrosine kinase receptors: VEGFR1, 2 and 3. Among other angiogenesis regulators, there are PDGF (platelet-derived growth factor), matrix metalloproteinases (MMPs), angiopoietins, and fibroblast growth factor (FGF). For most malignant tumors, angiogenesis is the initial process involved in tumor metastasis, which still represents one of the greatest clinical challenges associated with cancer [46].

In different cancer cell lines, fucoidans were able to inhibit the expression of the two key angiogenesis regulators VEGF and HIF-1α. For instance, fucoidan from *Fucus vesiculosus* (Sigma Aldrich, St. Louis, MO, USA) inhibited the mRNA expression of VEGF isoforms (VEGFA-VEGFD) in ovarian cancer cell lines (ES-2 and OV-90 cells) [21] (Table 1). Interestingly, the down-regulation of VEGFs by fucoidan was synergistically increased by the anticancer drugs paclitaxel and cisplatin. The expression of two other angiogenesis-related genes [i.e., KDR (kinase insert domain receptor) and FLT4 (FMS related tyrosine kinase 4)] was significantly decreased in ovarian cancer cells after treatment with fucoidan and chemotherapeutics. These findings, together with the reported ability of fucoidan to reduce proliferation, reactive oxygen species (ROS) generation and endoplasmic reticulum (ER) stress and induce the release of cytochrome c [21], disclose a multitarget anticancer mechanism for fucoidan in ovarian cancer cells.

Another preparation of fucoidan from *Fucus vesiculosus* showed pro-apoptotic and antiangiogenic effects in a human anaplastic thyroid cancer cell line (FTC133) [22] (Table 1). The mechanism behind the recorded antiangiogenic effect was the inhibition of VEGF and HIF-1α expression in hypoxia-like conditions. Tube formation and migration of thyroid cancer cells were both inhibited by fucoidan [22].

Similar results for fucoidan (Sigma Aldrich) were observed in triple-negative breast cancer cells MDA-MB-231 [30] (Table 1). In particular, in hypoxic microenvironment, fucoidan treatment significantly reduced cancer cell proliferation and the protein expression of HIF-1α and its target genes, such as TWIST, Snail, CAIX (carbonic anhydrase IX), and GLUT-1 (glucose transporter protein-1) [30]. These latter genes are EMT (epithelial-to-mesenchymal transition) regulators. EMT represents a biological program during which epithelial cells lose their cell identity and acquire a mesenchymal phenotype. EMT increases the processes of migration and invasion of cancer cells and is closely associated with tumor metastatization [47]. Through the inhibition of HIF-1α signaling, which can directly or indirectly affect EMT regulators, fucoidan inhibited EMT and metastasis in mammary cancer cells. The expression of the mesenchymal markers vimentin and N-cadherin was down-regulated by fucoidan, whereas the expression of the epithelial markers E-cadherin and ZO-1 (zonula occludens-1) was up-regulated [30]. Since cancer cells start detaching from the neighboring epithelial cells and invading the surrounding extracellular matrix by reducing E-cadherin expression, the up-regulation of E-cadherin triggered by fucoidan treatment hinders cancer cell migration. Similar results were obtained treating non-small cells lung cancer (NSCLC), A549 and H1650, with similar concentrations and time of treatment of fucoidan of unspecified origin [41] (Table 1). Fucoidan reduced NSCLC cell invasion via EMT inhibition, down-regulation of N-cadherin, and up-regulation of E-cadherin. Moreover, fucoidan blocked tumor angiogenesis via inhibition of the mTOR pathway and its downstream proteins p-S6K, p-P70S6K (ribosomal P70S6 kinase) and p-4EBP1 (translation initiation factor 4E binding protein 1) [41].

Similar results were recorded for fucoidan isolated from *Laminaria japonica* in triple-negative breast cancer cells (MDA-MB-231 and HCC1806 cells) [32] (Table 1). Fucoidan down-regulated the expression of proangiogenic factors, such as VEGFA, IGF-I (insulin-like growth factor-I), bFGF (fibroblast growth factors), MMP-2, and MMP-9, and suppressed the invasive and migratory capacity of both cell lines, as demonstrated via the transwell assay [32].

In vitro data demonstrated the antimigratory activity also for fucoidan from *Sargassum hemiphyllum* in human hepatocarcinoma cells [33] (Table 1). Among the signaling and molecular mechanisms regulating the metastatic process in human hepatocarcinoma, microRNAs have been demonstrated to play an important role. MicroRNA (miR) are small non-coding RNAs that negatively regulate gene expression of target messenger RNA [48]. In particular, decreased expression of miR-29 has been reported in many cancers, including hepatocarcinoma, and DNMT3B (DNA methyltransferase 3B), which is responsible for the silencing of tumor suppressor genes, has been identified as one of its downstream target [49]. In human hepatocellular carcinoma, the overexpression of DNMT3B was negatively associated with MTSS1 (metastasis suppressor 1) expression. Thus, the modulation of the axis miR-29b-DNMT3B-MTSS1 has been identified as a potential target to improve the treatment of hepatocarcinoma [33]. Fucoidan increased miR-29b and suppressed DNMT3B expression, which resulted in the up-regulation of MTSS1, inhibition of EMT, and prevention of ECM degradation. According to what was observed in the breast cancer model for fucoidan (Sigma Aldrich) [30], fucoidan from *Sargassum hemiphyllum* increased E-cadherin and decreased N-cadherin expression and, similarly to the fucoidan from *Laminaria japonica*, decreased the expression of MMP-2 and 9 and up-regulated TIMP-1 (tissue inhibitor of MMP-1) [33]. All these mechanisms contribute to inhibiting the invasion activity of hepatocarcinoma cells. The prevention of ECM degradation by fucoidan from *Sargassum hemiphyllum* was also mediated by the suppression of transforming growth factor β (TGF-β) signaling [33]. TGF-β has been shown to promote tumor invasion and metastasis by activating MMPs and Smad (suppressor of mothers against decapentaplegic). The mechanism of inhibition of TGF-β signaling was demonstrated for fucoidan from *Fucus vesiculosus* in breast cancer cells, where fucoidan boosted its ubiquitin-dependent proteasome-mediated degradation [50]. Based on these results, it is possible to speculate that the inhibition of miR-29b is associated with the down-regulation of TGF-β, and their suppression contributes to the antimetastatic effects of fucoidans.

Invadopodia are actin-like structures whose primary function is the degradation of ECM through the secretion of MMPs. They allow cancer cells to gain migratory and invasive abilities and join the blood stream [51]. Invadopodia maturation is a complex process, which involves several proteins. Src (steroid receptor coactivator), FAK (focal adhesion kinase), and cortactin are key proteins of the initiation stage of invadopodia formation. They mediate the interaction with integrins and adhesion to ECM and are crucial for the early invadopodia formation. In the assembly stage, Tks5 plays an important role in starting active polymerization, which requires N-WASP (neuronal Wiskott-Aldrich syndrome protein), ARP (actin related protein), and CDC42 (cell division control protein 42 homolog) proteins. Finally, the maturation stage involves the recruitment of MMPs. Fucoidan from *Sargassum fusiforme* [35] (Table 1) dose-dependently decreased the expression of invadopodia-related proteins, including Src, cortactin, N-WASP, ARP3, CDC42, MMP2, MT1-MMP (membrane-type 1 matrix metalloproteinase), and the targeting receptors integrin αV and β3 in HCCLM3 hepatocarcinoma cells [35]. Tumor tissues expressing αVβ3 tend to metastasize aggressively, thus targeting the formation of this complex will significantly block cancer metastasis. Taken together, those results show that fucoidan is able to counteract cancer metastatization interfering with all three stages of invadopodia formation and, thus, represents a promising antimetastatic compound.

Independently from the source of fucoidans, the polysaccharide exhibits antiangiogenic and antimigratory effects mainly mediated by the inhibition of VEGF, HIF-1α and EMT. The effects were observed in several cancer cell lines but at a very variable range of concentrations (5–30,000 µg/mL) (Table 1). This may suggest a different sensitivity of the cancer cell model and/or a different potency of fucoidans, depending on the source and fucose content.

#### 2.1.2. Endothelial Cells

Endothelial cells are widely used in the investigation of angiogenic and anti-angiogenic mechanisms [44]. Human umbilical vein endothelial cells (HUVEC) are the most used experimental model for exploring several cellular processes linked to angiogenesis, such as cell proliferation, cell cycle, tube formation, cell migration, and cell adhesion to matrix proteins. Low molecular weight fucoidan (LMWF) from *Sargassum hemiphyllum* was shown to inhibit hypoxia-induced angiogenesis and VEGF-induced capillary-tube-like structure formation in HUVEC cells [34] (Table 1). Interestingly, the ability of that LMWF to inhibit angiogenesis was not recorded in normoxic conditions, highlighting its hypoxia specific activity. In the same cell model, fucoidan from *Laminaria japonica* (100, 200, 500 µg/mL) suppressed tube formation [32] (Table 1). Moreover, HUVEC cells were treated with fucoidan before seeding with MDA-MB-231 cells expressing the green fluorescent protein (GFP), which enables to monitor tumor vasculature formation. Fucoidan blocked tumor-elicited tube formation, and the transwell assay showed that MDA-MB-231/GFP were inhibited in their process of adhesion and extravasation to vascular endothelial cells [32].

Key features of angiogenesis can be reproduced also using human aortic endothelial stem cells (HAEC). In particular, these cells were used to determine the effects of fucoidan from *Turbinaria conoides* on tube formation in the 3D matrigel matrix [38] (Table 1). Treatment with increasing concentrations of fucoidan determined a decline in HAEC tubule formation, which resulted completely suppressed at the highest tested concentration (100 µg/mL). Moreover, the analysis performed using AngioQuant, an automated image analysis tool for quantification of angiogenesis, showed a significant and concentration-dependent decrease in the total number of tubule junctions after fucoidan treatment [38].

Human pulmonary microvascular endothelial cells (HPMEC-ST1.6R cell line) were used as model to assess the antiangiogenic properties of fucoidan from *Fucus vesiculosus* [23]. The tube formation assay showed that fucoidan (500 µg/mL) administered during the seeding of endothelial cells inhibited the formation of tubular-like structures; fucoidan treatment after HPMEC-ST1.6R adhesion, migration, and organization completely disrupted the formation of new vessels. Interestingly, fucoidan treatment did not affect VEGF secretion, but significantly reduced the expression of PDGF and compromised the maturation of blood vessels [23]. Of note, at the tested concentration of 500 µg/mL, fucoidan showed antiangiogenic effect without affecting cell viability of HPMEC-ST1.6R cells, whereas at the same concentration it had cytotoxic effects on breast MDA-MB-231 cancer cells [23]. On the whole, these results would provide evidence that fucoidan exhibits its cytotoxic activity mainly towards cancer cells. However, whether fucoidans have a selective effect on cancer cells still seems questionable. Further studies are required to clarify this key aspect in the clinical translation perspective of fucoidan.

The molecular pathway regulating the inhibition of angiogenesis was investigated for fucoidan FP08S2 from *Sargassum fusiforme* in human HMEC-1 microvascular endothelial cells [36,37] (Table 1). FP08S2 (25–200 μg/mL) inhibited tube formation in a dose-dependent manner. A complete disruption of network formation of HMEC-1 cells could be observed at the highest tested concentration. Interestingly, the antiangiogenic activity of FP08S2 was compared with that of its desulphated derivative (FP08S2-DS). After FP08S2-DS treatment, no significant inhibition of tube formation was observed even at 200 μg/mL [37]. These results clearly indicate that sulfate groups play a crucial role in the anti-angiogenic activity of fucoidan. In the same experimental model, FP08S2 blocked the migration/invasion of HMEC-1 cells in a concentration dependent fashion [36,37]. The antimigratory and antiinvasive effects were recorded without affecting the viability of HMEC-1 cells. The mechanism behind the antiangiogenic effects of FP08S2 resides in its ability to bind to both VEGF-VEGFR2, thus not allowing their interaction. This blocks the VEGFR2-induced activation of the MAPK/PI3K signaling pathway and its downstream targets ERK and mTOR, resulting in angiogenesis inhibition.

Lymphangiogenesis, although less investigated than angiogenesis, contributes to tumor spread and metastasis. Fucoidan from *Undaria pinnatifida sporophylls* inhibited cell invasion and lymphatic metastasis in human and murine lymphatic endothelial cells (LEC) [39,40] (Table 1). The mechanism was investigated in human LEC, where fucoidan suppressed the NF-κB (nuclear factor kappa-light-chain-enhancer of activated B cells)/PI3K/Akt signaling pathway reducing the expression of the main regulators of lymphangiogenesis PROX1 (prospero homeobox protein 1) and VEGF3 [40].

Angiogenesis plays a key role in bone repair and development, as well as in tumor growth and formation of metastasis in bone tumors, such as osteosarcomas [24]. Co-culture systems of endothelial cells and circulating bone marrow cells represents a high relevant in vitro model to study both the physiological and pathological angiogenetic process in the bone [24]. In co-cultures of human peripheral blood-derived outgrowth endothelial cells (OECs) and human bone marrow-derived mesenchymal stem cells (MSCs) or human bone sarcoma cell line (MG63), treatment with fucoidan from *Fucus vesiculosus* (100 µg/mL) resulted in a significant decrease in length and area of angiogenic structure of OECs [24] (Table 1). The anti-angiogenic effects were associated with a decreased expression of VEGF and the pro-angiogenic protein angiopoietin-2. Moreover, SDF-1 (stromal derived factor-1), which plays a major role in the migration, recruitment, and retention of endothelial progenitor cells to hypoxic sites and contributes to neovascularization, was down-regulated in response to fucoidan treatment as well as its corresponding receptor CXCR4 (alpha-chemokine receptor specific for stromal-derived-factor-1) [24]. Taken together, these data indicate how the fucoidan-induced decrease in VEGF and SDF-1 protein expression is relevant in both the control of bone physiological angiogenesis and in the inhibition of osteosarcoma angiogenetic processes.

### 2.2. Antiangiogenic Effects of Fucoidans in Chick Embryo Chorioallantoic Membrane (CAM) Model

Among the in vivo models, CAM has been widely used to study molecules with angiogenic/anti-angiogenic activity. This model offers many advantages, such as low cost, simplicity, reliability, and reproducibility. Since chick embryos are not considered to be living animals until day 17 of development, CAM does not require ethics committee approval for animal experimentation [52]. A first study examined the antiangiogenic effects of fucoidan in fertilized chicken eggs treated with increasing concentrations of fucoidan from *Turbinaria conoides* [38] (Table 1). After 4 days from treatment, the analysis of the allantoic membrane showed a significant dose-dependent reduction in the total number of tubule junctions, thus neovascularization, compared with untreated samples [38]. In the same model, a potent and dose-dependent antiangiogenic effect was recorded also for FP08S2 [36] (Table 1), confirming the results obtained for FP08S2 in endothelial cells as reported above.

CAM is a useful model used to implant several tumor types as well as malignant cell lines and study their growth rate, angiogenic potential, and metastatic capability [52]. CAM allows to study either spontaneous or experimental metastasis in a short time: 7–8 days compared with 4–10 weeks for most typical murine models. A recent study investigated the antiangiogenic potential of crude fucoidan from *Fucus vesiculosus* in both CAM (to assess effects on vasculature) and CAM with tumor MDA-MB-231 cells onplantation (to assess effects in an in vivo tumor microenvironment) [23] (Table 1). After 7 days from fucoidan injection, a significant decrease in blood vessel formation was observed in CAM model when compared with the control (water), corroborating in vitro results obtained in HPMEC-ST1.6R cells. In the onplanted CAM model, fucoidan treatment did not induce a significant reduction in vessel formation, but a significant tumor shrinkage was observed compared with the control [23]. Even if a difference in terms of vascularization was not recorded in CAM onplanted with breast cancer cells, a lower expression of the endothelial cells marker lectin was demonstrated [23]. The decrease in lectin levels may be related to a decrease in blood vessel stability.

### 2.3. Antiangiogenic and Antimetastatic Effects of Fucoidans in Murine In Vivo Models

Many in vivo studies have been performed for different species of fucoidan in murine cancer models. The effects of fucoidan purified from *Fucus vesiculosus* (Sigma Aldrich) were investigated in lung [28], breast [29], colon [26,27], prostate [31], and multiple myeloma [25] cancer cells xenograft mice (Table 1). For example, in a mice model of murine Lewis lung adenocarcinoma, administration of fucoidan (1 or 3 mg/day by intragastric gavage) seven days prior to tumor cells inoculation significantly inhibited cancer cells spreading and proliferation, and down-regulated the expression of MMPs, NF-κB and VEGF [28]. Similar results were obtained in a study where fucoidan [5 or 10 mg/kg body weight (b.w.)] was intraperitoneally injected every two days for 20 days in mice transplanted with 4T1 breast cancer cells, which are able to generate lung metastases. Fucoidan significantly reduced VEGF protein tissue expression, tumor volume and weight and, as expected, decreased microvessel density in the harvested tumors [29]. Moreover, the number of lung metastases was significantly lower in fucoidan-treated mice compared to untreated mice, with a seven-fold decrease in the number of nodules at the highest tested concentration [29].

The same commercial preparation of fucoidan (Sigma Aldrich) was shown to reduce tumor growth and decrease angiogenesis in two different mice models xenografted with human colon HT29 cells [26,27], following similar modality of treatment compared to the aforementioned study [29] (Table 1). In both colon cancer studies, the antiangiogenic effects of fucoidan were driven by the down-regulation of VEGF and the decreased expression of the marker of vascular density CD31 (cluster of differentiation 31). The effect of fucoidan was studied in synergy with the prion silencing in HT29 cancer cells. The prion protein PrP^c^ is associated with the growth of several type of tumors, including colon cancer [53]. The silencing of the prion protein induced synergistic effects with fucoidan treatment, improving both antiangiogenic and antiproliferative effects [26]. However, further studies are necessary to clarify how the silencing of PrP^c^ affects fucoidan anticancer effects.

The antiangiogenic effects of fucoidan were reported also after oral administration. In a prostate cancer model, the oral administration of fucoidan (Sigma Aldrich) (20 mg/kg b.w. for 28 days) significantly hindered tumor growth and angiogenesis [31]. In particular, a decrease in hemoglobin content and in the expression of CD31 and CD105, markers of angiogenesis in human malignancies [54], were recorded in the tumor tissue. STAT3 (signal transducer and activator 3) is usually phosphorylated by the receptor activated Janus kinase (JAK), and its abnormal activation is usually associated with tumor angiogenesis and prostate cancer progression [55]. Fucoidan treatment reduced the activation of both JAK and STAT3 in prostate tumor tissue and also reduced the expression of its downstream targets regulating angiogenesis, such as VEGF [31].

Taken together, in vivo results agree with the in vitro evidence that VEGF is a key molecular target for the anticancer activity of fucoidan from *Fucus vesiculosus*.

In vivo evidence on the antiangiogenic and antimetastatic activity of fucoidan have been collected also for fucoidan from *Sargassum fusiforme* [35,36] (Table 1). It showed antimetastatic effects, reducing the number of lung metastatic foci in nude mice xenografted with HCCMLM3 hepatocellular carcinoma cells (1 g/kg orally administered for 21 days) [35]. Moreover, the preparation FP08S2 from *Saragassum fusiforme* inhibited microvessel formation in a murine xenograft model of lung cancer (up to 10 mg/kg via tail vein injection every day for 27 days) [36]. The histological analysis of the tissue revealed a significant inhibition of CD31 expression. The recorded inhibition of tumor growth was at least partially due to the anti-angiogenic effect of fucoidan. Although the dose and the way of administration of the two preparations of fucoidan from *Sargassum fusiforme* were markedly different, in both cases fucoidan significantly reduced tumor size and weight.

In contradiction to the huge number of results reported in Table 1 and described so far, the anticancer activity of a fucoidan of not specified origin provided by Ze Lang Nanjing Medical Technology Co., Ltd. (Nanjing, China) seems to be independent from the antiangiogenic activity of the polysaccharide, as shown in a model of human hepatocarcinoma [56]. In particular, in female nu/nu nude mice xenografted with Bel-7402 hepatoma cells, the cavitas abdominalis injection of 20 or 200 mg/kg b.w. fucoidan once a day for 25 days reduced tumor volume and weight. However, fucoidan had no effects on the expression of bFGF and VEGF in tumor tissue and on microvascular density of tumors compared to control [56], highlighting an anticancer mechanism independent from its antiangiogenic potential. Moreover, fucoidan exhibited low affinity for bFGF and could not block the binding of bFGF to heparan sulphate, which is responsible for the release of pro-angiogenic factors [57]. Unfortunately, the uncharacterized source and structure of the fucoidan do not allow us to draw a conclusion with any certainty. The antiangiogenic activity of fucoidan from *Laminaria Japonica* was also investigated in a diethyl nitrosamine (DEN)-induced hepatocarcinoma rat model in combination with the two anti-angiogenic drugs avastin and sorafenib [58]. Fucoidan alone did not significantly decrease the expression of AFP (alpha fetoprotein), a key marker of tumor burden and prognosis, VEGF or VEGFR, although a reduction in their levels was observed in combination with the anti-angiogenic drugs [58]. These results do not show a clear advantage derived from fucoidan administration.

MW and sulfation degree are known to influence the antiangiogenic activity of this polysaccharide, as discussed above. The antiangiogenetic potency is higher for fucoidan with higher degree of sulfation and higher MW [59]; fucoidans of low MW may even act as pro-angiogenic agents [17,60]. Since the structure of fucoidan is not fully described and characterized in several papers, it is not possible to clearly define which structural characteristics of fucoidan determine its antiangiogenetic activity.

The preclinical potential of fucoidan as an antiangiogenic agent was also explored for a nanoparticle formulation. In particular, newly designed fucoidan coated-manganese dioxides nanoparticles (Fuco-MnO_2_-NPs) were investigated with the aim to conjugate the bioactive fucoidan with the oxygen generating MnO_2_-nanoparticles in a model of pancreatic tumor [43] (Table 1). Radiotherapy still represents a good treatment opportunity to shrink pancreatic tumors before surgical resection; however, the radiosensitivity of the tumor is strongly influenced by the presence of oxygen and the presence of a hypoxic core often compromises its efficacy. Fuco-MnO_2_-NPs were designed to meet the urgent need for novel hypoxic radiosensitizer. Fucoidan can contribute with (i) its pharmacological activity that could be synergistic with that of radiation therapy and with the oxidant activity of MnO_2_-NPs, and (ii) its demonstrated ability to reduce the toxicity of metallic nanoparticles [61]. The combination of intratumoral injection of Fuco-MnO_2_-NPs and radiation therapy delayed tumor growth compared to radiotherapy alone. Moreover, the analysis of tumor tissue showed a reduction in phospho-VEGFR2, supported by a decrease in HIF-1α and CD31 expression, indicating that Fuco-MnO_2_-NPs significantly compromised tumor angiogenesis [43,62].

### 2.4. Antiangiogenic and Antimetastatic Effects of Fucoidans in the Zebrafish Model

Over the last decade, zebrafish have emerged as a promising experimental model to study tumor angiogenesis and metastasis. The zebrafish embryos vasculature shows a strong similarity to vascularization in humans and grows rapidly. Thus, a single blood circulatory loop in zebrafish is fully developed in 24 h post-fertilization [63,64]. Moreover, the vascular endothelial cells can be stained with a fluorescent protein to monitor the neovascularization process in the tumor microenvironment and the transparent body of zebrafish embryos allows non-invasive imaging and analysis. According to the same principle, in the zebrafish model it is possible to visualize the dissemination of fluorescently labelled tumor cells and invasion in surrounding tissues [63,64].

The antiangiogenic activity of fucoidan has been investigated taking advantage of the Tg(flk1:EGFP) zebrafish embryos model, a transgenic fish line with a tissue-specific expression of GFP in the vasculature, which allows to monitor individual cell growth and vessel formation. In particular, treatment with increasing concentrations of fucoidan from *Laminaria japonica* for 48 h blocked angiogenesis and impaired the vascular development up to 75% at the highest tested concentration (2000 µg/mL) [32] (Table 1). In the same transgenic zebrafish model, fucoidan from *Fucus vesciculosus* (300 µg/mL) disrupted vascular development after 48 h from treatment. Moreover, the expression of the angiogenesis-related VEGFA-, VEGFC-, FLT1-, FLT4-, KDR-, and KDR-like genes dramatically decreased in fucoidan-treated transgenic zebrafish embryos [21] (Table 1), confirming the inhibitory activity of angiogenesis by fucoidan both at cellular and molecular levels.

To monitor the ability of fucoidan to inhibit tumor metastatization, GFP-expressing MDA-MB-231 cells were pretreated with fucoidan from *Laminaria japonica* for 24 h and injected into the perivitelline cavity of wild-type zebrafish embryos [32] (Table 1). Two days after the injection, embryos were imaged, and distant micrometastases were exhibited in 60% of control and only in 18% of fucoidan-treated tumor cells [32], suggesting that fucoidan significantly attenuated the metastatic capability of triple-negative breast cancer cells.

The anticancer efficacy of fucoidan was investigated in another model of onplanted xenograft model. In particular, fucoidan from *Fucus vesciculosus* was used to pre-treat ovarian cancer cells, which were then injected in the zebrafish [21] (Table 1). A significant decrease in tumor size was recorded compared to control [21].

The lysine-deficient protein kinase-1 (WNK1) is a critical protein in both embryonic- and cancer-induced angiogenesis and is considered a potential therapeutic target to contrast cancer progression. Two downstream effectors of WNK1-mediated angiogenesis are the oxidative stress responsive 1 (OSR1) and the protein phosphatase 2A (PPP2R1A) proteins. Up-regulation of WNK1 and OSR1 in endothelial cells favors the process of neo-angiogenesis, and the axis WNK1-ORSR1-PPP2R1A was demonstrated to play a critical role in both endothelial (HUVEC) and hepatoma (HepG2) cells during angiogenesis and cell migration [42]. The zebrafish model was used in a very recent study to demonstrate how the co-administration of oligo-fucoidan could improve the anti-cancer efficacy of WNK463 (a WNK1 inhibitor) and rafoxanide (an OSR1 inhibitor) in advanced hepatocellular carcinoma [42] (Table 1). The main mechanism responsible for the anticancer activity of fucoidan in association with WNK463 and rafoxanide has been ascribed to its ability to counteract the inflammatory response evoked by the aforementioned inhibitors [42]. However, it is possible to hypothesize that the antiangiogenic and antimetastatic activity of fucoidan may contribute to the recorded anticancer effects.

## 3. Clinical Studies on Fucoidans

The anticancer activity of fucoidans observed in preclinical studies is attractive to pave the way for the discovery of new oncological strategies. To date, fucoidans have been investigated as dietary supplements in combination with traditional chemotherapeutics, but their efficacy as monotherapy to fight cancer has not yet been assessed. An insight into the most relevant clinical evidence of the last 10 years is presented below.

A randomized, double-blind, controlled trial investigated the clinical benefit of the low MW fucoidan (0.80 kDa) extracted from *Sargassum hemiphyllum* and widely studied in vitro, as reported above. Fucoidan was used as supplemental therapy in patients with metastatic colorectal cancer [65]. More in details, 28 patients were randomly assigned to treatment group and 26 to control group. Both groups received biweekly folinic acid, 5-fluorouracil, and irinotecan plus bevacizumab as first-line chemotherapy. The patients of the treatment group orally took 4 g of fucoidan twice a day while control patients received 4 g of cellulose powder twice a day as placebo. The duration of the intervention was six months. In the treatment group, the disease control rate, defined as the sum of the complete response, partial response, and stable disease, was significantly higher than in the control group (92.8% vs. 69.2%, respectively *p* = 0.026) in a median follow-up period of 11.5 months. Moreover, although the differences were not significant compared to the control group, the treatment group showed a better overall survival rate (18.04 ± 0.91 vs. 12.96 ± 0.83 months) and progression-free survival rate (15.93 ± 1.20 vs. 10.80 ± 1.06 months). Fucoidan did not seem to affect blood biochemical markers, and the grading of leukopenia, anemia, and thrombocytopenia was similar in both groups. During the follow-up, the quality of life of patients was also assessed using a questionnaire (EORTC QLQ-CR29). The two groups showed a similar trend as regards the limitation of daily activities, limitation of walking, anxiety, fatigue, and wellness. Although the treatment group was better in terms of doing hobbies, trouble sleeping and depression, the differences were not significant compared to the control group. Since the identification of therapeutic strategies for patients with advanced cancer is an urgent concern, the set of results obtained can be “a starting point” for the future development of fucoidan in combined chemotherapy.

In 2020, a phase II randomized, double-blind study was started to evaluate the clinical effects and safety of a combination of fucoidan, chemotherapy, and radiation therapy in the treatment of cancer patients with stage III/IV of head and neck squamous cell carcinoma (NCT04597476). The 119 patients enrolled in the study were randomized in a 1:1 ratio to receive orally either fucoidan (4.4 g, oral administration twice a day) or placebo (potato starch 4.4 g twice a day) over a 24-week period in combination with chemotherapy and radiation therapy. The disease-free survival of patients is the primary outcome of the study, while the evaluation of pain and the determination of the disease control rate are measured as secondary outcomes. Moreover, the study monitors the incidence of treatment-emergent adverse events and evaluates the quality of life of patients using EuroQol five dimensions questionnaire. The estimated study completion date is scheduled for 25 March 2023; therefore, the results are not yet available.

In the last 5 years, two other clinical trials have been planned to assess the benefits of fucoidan as supplemental therapy in advanced cancer patients. However, the status of both studies is unknown, and the results have not yet been reported. In particular, one phase II randomized, double-blind, controlled trial was performed to investigate the efficacy of oligo-fucoidan dietary supplement (MW ranged from 0.50 to 0.80 kDa; 4.4 g, oral administration, twice a day) in terms of disease control rate in patients with advanced hepatocellular carcinoma (NCT04066660). The other double-blind, randomized, placebo-controlled study aims to evaluate the efficacy of fucoidan to improve the quality of life in patients with locally advanced rectal cancer who receive combined radio-chemotherapy before surgery (NCT04342949).

The complex relationship between inflammation and tumor development and metastatization has been well demonstrated. Counteracting inflammation in cancer patients could be useful to alleviate cancer-related symptoms, but also to improve the clinical efficacy of chemotherapy [66,67]. An open-label, single-arm clinical study was undertaken in 20 advanced cancer patients to assess the efficacy of fucoidan on inflammation in relation to the quality of life of patients [68]. Although patients were heterogeneous due to the origin of their primary tumor, they all had distant metastases, and 90% of them had received standard chemotherapy treatment for advanced cancer before fucoidan administration. Each patient received 400 mL/day of fucoidan for 4 weeks. The aqueous solution contained 10 mg/mL of Power Fucoidan extracted from *Cladosiphon novae-caledoniae* (Daiichi Sangyo, Cp Ltd., Osaka, Japan). The molecular composition of Power Fucoidan included 72% digested low MW fractions (MW smaller than 0.50 kDa), and 28% non-digested fraction (with a peak MW of 800 kDa). Proinflammatory cytokines, such us interleukin-1β (IL-1β), IL-6, IL-8, and tumor necrosis factor-α (TNF-α), were significantly decreased after 2 weeks of fucoidan intake. Notably, the reduction in IL-8 was significantly associated with the increase in overall survival rate of cancer patients. On the other hand, no significant changes were observed in the quality of life of patients during the period of the study. The ability of fucoidan to improve inflammation status could help the design of new approaches to prevent cancer recurrence and metastasis. However, these findings should be considered with caution, as the results were obtained in a small single group study. Future randomized controlled trials with a larger sample size are needed to confirm the benefit of the anti-inflammatory effects of fucoidan in cancer patients.

One of the challenges of cancer therapy is to identify effective strategies to counteract the toxicity of radiotherapy and chemotherapy.

Cisplatin is a well-known chemotherapy drug for the treatment of different types of cancer. However, the administration of cisplatin is often hampered by the occurrence of serious adverse effects in cancer patients [69]. One study investigated the efficacy of fucoidan to reduce the adverse effects associated with cisplatin treatment in 24 patients with unresectable advanced gastric cancer [70]. The patients were randomly assigned to the fucoidan-treated group (*n* = 12) or the control group without fucoidan treatment (*n* = 12). Cisplatin treatment was administered to all 24 participants. The patients of the fucoidan group also took 150 mL/day of liquid that contained 4.05 g of a high MW fucoidan derived from *Cladosiphon okamuranus* (Okinawa mozuko, Marine products Kinuraya Co., Ltd., Tottori, Japan) for six months after the beginning of chemotherapy. The median follow-up period of the 24 patients was 10 months (range 3–17 months), and all 12 patients completed fucoidan treatment safely. Fucoidan suppressed the occurrence of diarrhea and reduced general fatigue during chemotherapy. Moreover, fucoidan maintained a favorable nutritional status of patients during chemotherapy and prevented the deterioration of prognostic nutritional index due to cisplatin (PNI: 47.6 ± 6.1 vs. 39.4 ± 8.2; *p*: 0.028). Therefore, the fucoidan group could continue the chemotherapy for a longer period (7.4 months vs. 4.6 months; *p*: 0.044), and the mean survival time of the 12 patients receiving fucoidan supplement was significantly better than that of the 12 control patients (12 months vs. 8 months, *p*: 0.039).

More recently, in 2022, recruitment started for two clinical trials aimed at investigating the benefits of dietary supplementation with fucoidan in cancer patients. A randomized-parallel study will test the effect of oligo-fucoidan to mitigate the lung damage that can occur as a side effect of radiation therapy in lung cancer patients (NCT05616507). The other randomized-parallel study will evaluate the ability of oligo-fucoidan to improve cachexia or sarcopenia in cancer patients (NCT05623852). The data collection of the two clinical trials is expected to be completed in 2024. The information that will be reached can be pivotal to clarify the benefits of fucoidans on reducing adverse effects during radio-chemotherapy and to promote its development as a complementary therapy in oncology.

A molecule administered as a complementary chemotherapy or dietary supplement in cancer patients should not reduce the bioavailability of other co-administered chemotherapeutic agents. Information on potential pharmacokinetic interactions between fucoidans and conventional anticancer therapies is lacking. To the best of our knowledge, only an open label non-crossover study assessed the effect of co-administration of *Undaria pinnatifida*-derived fucoidan on pharmacokinetics of letrozole or tamoxifen in patients with breast cancer [71]. Overall, 20 female patients were enrolled in the study: 10 received letrozole and 10 received tamoxifen. All patients orally took fucoidan (Maritech extract, Marimova Pty Ltd., Hobart, Australia) for a period of 3 weeks (500 mg twice daily). HPLC analysis performed at baseline and after 3 weeks showed that co-administration of fucoidan did not affect the steady-state plasma concentration of letrozole, tamoxifen, or tamoxifen metabolites. In addition, no adverse effects of fucoidan were reported over the study period. Future well-conducted randomized control trials are necessary for a comprehensive assessment of possible pharmacokinetic interactions and clinical benefits of fucoidan treatment combined with various classes of anticancer drugs.

Taken together, the available findings indicate that oral intake of fucoidan as dietary supplement in combination with conventional adjuvant chemotherapy can prolong survival time, decrease some adverse effects (e.g., fatigue) and ameliorate the inflammatory state in patients with advanced cancer or metastasis. On the contrary, it is not possible to establish the benefit of fucoidan on the quality of life of patients. However, the understanding of the effectiveness of fucoidan in cancer therapy is far from being clear. Evidence was observed in relatively small groups and enrolled patients were mainly suffering from advanced gastrointestinal cancer. Moreover, the source and formulation of the administered fucoidan were heterogeneous (e.g., different MW). Future randomized controlled trials with a larger sample, recruiting patients with different types and stages of cancer are necessary to assess the efficacy of fucoidans in clinical oncology.

## 4. Pharmacokinetics of Fucoidans

Knowledge of the pharmacokinetic profile of a drug candidate is crucial to ameliorate success rates and decrease candidate attrition during the drug development process. Pharmacokinetic studies evaluate how the route of administration affects the processes of absorption, metabolism, and excretion of a drug. This evidence is fundamental for the selection of the recommended dosage and for the optimal efficacy of drugs. The pharmacokinetics of fucoidans with different MW and obtained from various sources has been studied in various animal models in relation to the different routes of administration. Shikov and colleagues recently reviewed and summarized their pharmacokinetic parameters [72]. Since the clinical studies discussed above provide the systemic effects of fucoidan after oral administration in cancer patients, here we report a brief and updated focus on the pharmacokinetics of fucoidans in animals and in humans after oral intake.

Zhang and colleagues [73] compared the pharmacokinetics of fucoidan (MW: 7.1 kDa) from *Laminaria japonica* in rabbit after intravenous injection (50 mg/kg b.w.) and oral administration (200 mg/kg b.w.). The amount of fucoidan was determined in serum by HPLC with post-column fluorescent derivatization. Following intravenous injection, fucoidan reached a maximum plasmatic concentration (C_max_) of 110.53 μg/mL in 5 min and it was measurable up to 6 h. Moreover, the half-time (T_1/2_) of distribution phase was 11.24 min and the T_1/2_ of elimination was 98.20 min. On the other hand, the bioavailability of fucoidan within 24 h of oral administration was very low, and fucoidan was determined only after 2 h from ingestion. The different pharmacokinetic profiles suggest that fucoidan is rapidly degraded following oral administration and its metabolites are not determined in serum by the applied HPLC method. In addition, it may be possible that fucoidan is quickly eliminated from the gastro-enteric tract and not absorbed into the blood.

The influence of MW on the pharmacokinetic profile of fucoidans was assessed in rat models [74]. Low MW (7.6 kDa) and medium MW (35 kDa) fucoidans from *Laminaria japonica* were administered orally to male rats at doses of 200, 400, and 800 mg/kg b.w. The monosaccharide composition and fucose content were determined in rats’ plasma and urine samples within 48 h via reverse-phase HPLC. The results indicated that low MW may improve intestinal absorption and bioavailability of fucoidan. In fact, after administration of 800 mg/kg, the C_max_ levels of low MW fucoidan in blood were 151.7 μg/mL T_max_ 15 h (time at which the C_max_ is observed), whereas C_max_ was 131.8 μg/mL for medium MW at T_max_ 25 h, suggesting higher bioavailability for the low MW fucoidan.

Since fucoidans are a group of complex sulphated polysaccharides, the interest in evaluating the uptake and distribution of fucoidans with high MW has grown over the last years. Pozharitska and colleagues [75] investigated the bioavailability and tissue distribution of fucoidan with high MW (735 kDa) from *Fucus vesiculosus* in male rats after oral administration at the dose of 100 mg/kg b.w. Due to the heparin-like structure of fucoidan, the method of fucoidan detection used in the study was the analysis of the plasma anti-activated factor X (anti Xa) activity, which is normally used to determine the anticoagulant level of enoxaparin in blood and tissues [75]. Fucoidan was detected in plasma 30 min after oral administration and reached the C_max_ of 0.125 μg/mL in 4 h with a T_1/2_ elimination of 3.44 h. Interestingly, the evaluation of the area under the curve (AUC_0–t_, representing the total exposure to a drug integrated over the time) showed that fucoidan preferentially accumulates in the organs with filtering function, such as kidney (AUC_0–t_ = 10.74 μg h/g; C_max_ = 1.23 μg/g after 5 h), spleen (AUC_0–t_ = 6.89 μg h/g; C_max_ = 0.78 μg/g after 3 h), and liver (AUC_0–t_ = 3.26, μg h/g; C_max_ = 0.53 μg/g after 2 h). A similar tissue distribution was also observed after the injection of 50 mg/kg b.w. of fluorescein isothiocyanate (FITC)-labelled fucoidan (MW = 107.8 kDa) from *Fucus vesiculosus* into the tail vein of mice [76]. FITC-fucoidan reached C_max_ of 66.37 μg/g after 30 min, then decreased quickly, and after 4 h the compound was no longer measurable in the blood. The FITC-fucoidan was detected in the liver, spleen, lung, and kidney within 24 h, and the highest concentration was always found in the kidney tissue. Notably, the level of FITC-fucoidan in the kidney tissue reached 1092.31 µg/g after 30 min. These findings agree with the study of Pozharitska [75] and confirm the ability of the kidney to uptake and accumulate fucoidans.

The mechanism underlying the intestinal absorption of fucoidans has been characterized through in vitro assays. Nagamine et al. [77] showed that fucoidan from *Cladosiphon okamuranus* (South Product Co., Uruma, Japan) penetrated through the Caco-2 cells monolayer by active transport and achieved the maximum concentration after 1 h of treatment, followed by a rapid reduction in the uptake. Moreover, Bai and colleagues demonstrated the involvement of the clathrin endocytic pathway in the absorption and transport of FITC-fucoidan in Caco-2 cell line, confirming its ability to cross the intestinal barrier [76].

The set of results obtained in preclinical studies indicate that fucoidans with different MW can be absorbed after oral administration and distributed in tissue and organs. However, since fucoidans are high MW polysaccharides, it is fair to wonder whether in humans they can cross the intestinal barrier and circulate in the blood after oral ingestion. Clinical studies evaluating the effects of fucoidans as dietary supplements in patients with advanced cancer do not report data on the absorption and bioavailability of the fucoidans tested. This makes it difficult to assess the correlation between fucoidan’s pharmacokinetic profile and fucoidan-induced responses. Nevertheless, in the last decade, two studies on Japanese volunteers have tried to fill the gap regarding the absorption of fucoidans after oral intake [78,79]. In the first study, 48 volunteers (34 males and 14 females) living in Okinawa Prefecture, and 38 volunteers (17 males and 21 females) living in Gunma Prefecture (with no sea coast) ingested 100 g of Okinawa mozuko from *Cladosiphosn okamuranu* containing 1 g of fucoidan [78]. Urine was collected 0, 3, 6, and 9 h after mozuku intake. Fucoidan concentrations in urine were measured using ELISA [77]. Although a marked difference in fucoidan urinary concentrations was determined among volunteers, the set of results suggests that fucoidan can be absorbed after mozuko digestion. One year later, the study was expanded by enrolling 396 volunteers (227 males and 169 females) [79]. Depending on the place of residence, the volunteers were divided into two groups: (1) living in Okinawa prefecture (68%); (2) living outside Okinawa prefecture (32%). Notably, the habit of eating mozuku was more frequent in volunteers living in Okinawa prefecture than those living outside. Each volunteer took 3 g of mozuku fucoidan orally. The concentrations of fucoidan were determined in urine [77] at 0, 3, 6, 9 h after ingestion of fucoidan using ELISA. The urinary excretion of fucoidan was detected in 97% of volunteers, indicating fucoidan absorption in the gastrointestinal tract. Interestingly, the maximum value of urinary fucoidan was higher in volunteers living in Okinawa prefecture (332.3 ± 357.6 µg/g_creatinine_) than that of volunteers living outside (240.1 ± 302.4 µg/g_creatinine_). In addition, multiple regression analysis showed that the residence in Okinawa prefecture was a main significant factor contributing to the excretion of fucoidan. Since the habit of eating mozuku was higher in the group living in the Okinawa Prefecture, it is possible to hypothesize that the habit of eating mozuku helps the intestinal absorption of fucoidan. Although these findings confirmed the gastrointestinal absorption of fucoidan in humans, the Authors did not report data on plasma concentrations, bioavailability, and T_1/2_ of elimination. This precludes the possibility to define the pharmacokinetic profile of fucoidan in humans after oral intake.

Taken together, the results obtained in animal models after oral ingestion showed intestinal absorption and distribution in organs and tissues of fucoidans. Otherwise, the key pharmacokinetic parameters of fucoidans have not yet been clarified and reported in humans. Furthermore, the metabolism of fucoidans should be addressed in future pharmacokinetic studies to better understand their pharmacological activities and clinical potential.

## 5. Challenges and Future Perspectives

Despite the preclinical evidence of anticancer activities [80], the progress of fucoidans in tumor therapy is full of challenges. First of all, the knowledge of composition and structure of fucoidans is of crucial importance for its bioactivity. In particular, MW, nature of sugars, sulfate content and sulfate group positioning impact on its bioactivity [81].

Fucoidans from *Fucus vesiculosus* and *Undaria pinnatifida* are recognized as GRAS (Generally Recognized As Safe) by the USA Food and Drug Administration (FDA), based on their favorable toxicological profile, and used as a functional food [82]. Fucoidans are considered safe, due to their long history of traditional use in Chinese medicine [83]. One of the most used and consumed fucoidan is that one extracted from Okinawa mozuku. Its acute toxicity was tested in Wistar rats. No significant toxicological changes were recorded for fucoidan up to 600 mg/kg b.w. per day. Only with higher concentrations (1200 mg/kg b.w. per day), clotting time was significantly prolonged, an effect due to fucoidans heparin-like structure. Other signs of toxicities were not observed [84]. In the same rat model, sub-chronic (6 months) administration of fucoidan up to 300 mg/kg b.w. per day by oral gavage showed no significant toxicological effects [85]. Only when the dose was increased to 900 and 2500 mg/kg b.w. per day, the clotting time was significantly prolonged [85]. In a more recent study, no significant side effect was recorded up to 1000 mg/kg b.w. per day after oral administration of fucoidan from *Undaria pinnatifida* in Sprague Dawley rats for 28 days. At the tested concentration of 2000 mg/kg, a slight but significant increase in ALT (alanine transaminase) plasma level was recorded [86]. Taken together, the animal studies reported above indicate that the anticoagulant activity of fucoidans should be carefully considered as an adverse effect of its application as an anticancer therapy.

Encouraging results on the safety of fucoidans in humans are reported in a clinical trial that evaluated the improvement of the metabolic profile of 57 patients with non-alcoholic fatty liver disease (NAFLD) after ingestion of 275 mg oligo-fucoidan + 275 mg HS Fucoxanthin (NCT02875392) with a follow up of 6 months. No serious adverse effects were recorded in treated patients. Another double-blind, randomized, controlled trial showed that the treatment with *Fucus vesiculosus* extract (85% fucoidan) was safe and well tolerated in osteoarthritis patients. In particular, the fucoidan dose of 300 mg/day for 12 weeks did not induce any significant changes in the blood markers linked to haemopoietic, hepatic, and renal system toxicity or any treatment-related adverse events [87].

In the future perspective of fucoidans as combinatorial treatment in cancer therapy, pharmacokinetics represents an urgent task to be addressed. Human studies on healthy volunteers have been conducted among the Japanese population, who are in the habit of consuming brown seaweed [79]. Algae feeding is associated with the concomitant ingestion of marine bacteria that adapt to live in gut microbiota. These bacteria synthesize enzymes capable of metabolizing polysaccharides such as fucoidans and favoring their intestinal absorption [88]. An open question is about the absorption and bioavailability of fucoidans in the general population not used to algae consumption. Similarly, most clinical cancer studies have been performed in the Asian population who are used to algae consumption. Thus, it is not easy to predict whether the effects of fucoidan ingestion in cancer patients are related to the activity of specific enzymes present in the microbiota. The results of a planned clinical trial (status: unknown) focused on the biodistribution and tolerance of radiolabeled fucoidan (99 m Tc) (NCT03422055) will certainly help us to gain insight into the toxicological and pharmacokinetic profile of fucoidans in human beings.

## 6. Conclusions

**Figure 2:**
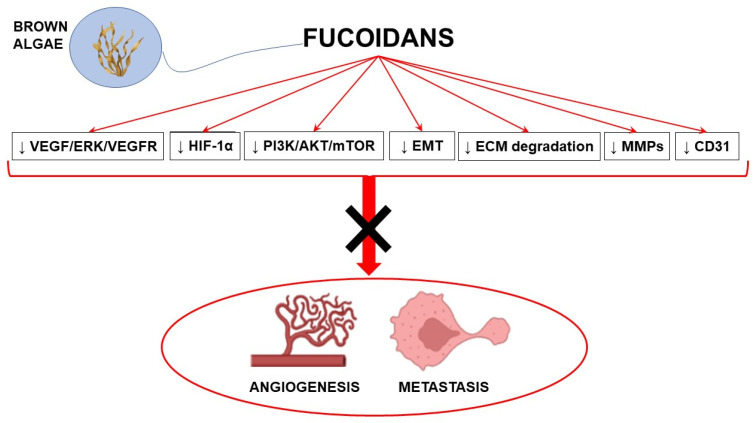
Schematic representation of the main molecular pathways modulated by fucoidans to induce antiangiogenic and antimetastatic effects (with the use of Biorender.com, accessed on 23 March 2023). ↓ inhibition; AKT: protein kinase B; CD31: cluster of differentiation 31; ECM: extracellular matrix; EMT: epithelial mesenchymal transition; ERK: extracellular signal-regulated kinase; HIF-1α: hypoxia-inducible factor 1α; MMPs: matrix metalloproteinases; mTOR: mammalian target of rapamycin; PI3K: phosphatidylinositol-3 kinase; VEGF: vascular endothelial growth factor; VEGFR: VEGF receptor.

Antiangiogenic agents may substantially contribute to the achievement of significant advances in cancer treatment. Most antiangiogenic drugs target the VEGF pathway; however, multitarget agents able to interact with multiple anticancer mechanisms represent a promising therapeutic approach. In this context, fucoidans with their complex antiangiogenic profile may represent valuable candidates in combination with conventional anticancer strategies to counteract cancer development. Preclinical evidence strongly supports the antiangiogenic and antimetastatic properties of fucoidans, although its mechanisms of action are not completely understood and highly depend on the source, the structure, and other characteristics of fucoidans (Figure 2).

To date, the most credited use of fucoidans in cancer management seems to be as diet supplements in combination with chemotherapy. Only when supported by clinical data and after a careful structure–activity characterization, can fucoidans from marine algae emerge as a promising anticancer strategy.

## Figures and Tables

**Table 1:** Antiangiogenic and antimetastatic effects of fucoidans.

Fucoidan	Cancer Model	Experimental Model	Antiangiogenic and Antimetastatic Mechanisms	References
Fucoidan (from *Fucus vesiculosus*) (Sigma Aldrich, St. Louis, MO, USA)	Human ovarian cancer	*Vitro*: ES-2 and OV-90 cell lines (300 µg/mL for 24/48 h) *Vivo*: Embryos of transgenic zebrafish Tg(fli1:EGFP) (300 µg/mL for 48 h) or zebrafish xenograft model injected with fucoidan pre-treated ES-2 and OV-90 cells	↓ mRNA expression of VEGFs (VEGFA-VEGFD), FLT4, KDR ↓ VEGFA, VEGFC, FLT1, FLT4, KDR, KDRL	[21]
Fucoidan (from *Fucus vesiculosus*)	Human anaplastic thyroid cancer	*Vitro*: FTC133 cell line (10 µM)	↓ HIF-1α and VEGF in hypoxic-like conditions ↓ Tube formation and the migration of FTC133 cells	[22]
Crude fucoidan (from *Fucus vesiculosus*) (Sigma Aldrich)	Human breast cancer	*Vitro*: MDA-MB-231 cell line, HPMEC-ST1.6R cells (500 µg/mL crude fucoidan for 4, 8, 12 or 16 h) *Vivo:* CAM without tumor (3 or 7 days after 0.5 mg/mL fucoidan injection) or CAM with tumor MDA-MB-231 cells onplantation (4 or 8 days after 0.5 mg/mL fucoidan injection)	↓ PDGF ↓ Migration and formation of new vessel ↓ Blood vessel maturation Disruption of pre-formed tubular-like structures ↓ Blood vessel stability (as demonstrated by the reduction in lectin expression)	[23]
Crude fucoidan (from *Fucus vesiculosus*) (Sigma Aldrich)	Human osteosarcoma	*Vitro:* OECs in co-culture with MSCs or MG63 (100–500 μg/mL)	↓ VEGF and SDF-1/CXCR4, collagen type 1 and angiopoietin-1 ↓ angiopoietin-2	[24]
Fucoidan (from *Fucus vesiculosus*) (Sigma Aldrich)	Human multiple myeloma	*Vitro:* RPMI-8226 and U266 cell lines (25–200 μg/mL for 72 h) HUVECs (incubated with conditioned medium from fucoidan-pretreated RPMI-8226 or U266 cells for 6 h) *Vivo*: Multiple myeloma cells xenograft NOD/SCID female mice (intraperitoneal injection with 10 or 50 mg/kg fucoidan every two days for 3 weeks)	↓ VEGF and HIF-1α both in hypoxic and normoxic conditions ↓ Tube structures and HUVECs migration ↓ p-AKT and p-ERK1/2 ↓ Angiogenesis and CD34+ vessels ↓ Microvessels density	[25]
Fucoidan (from *Fucus vesiculosus*) (Sigma Aldrich)	Human colon cancer	*Vitro:* Prion silenced HT29 cell line (200 μg/mL fucoidan for 48 h) *Vivo*: HT29 cancer cells xenograft Balb/c mice (5 mg/kg fucoidan intraperitoneally injected every 2 days for a total of nine administrations)	↓ Angiogenesis; ↓ Migration ↓ Angiogenesis, VEGF and CD31	[26]
Fucoidan (from *Fucus vesiculosus*) (Sigma Aldrich)	Human colon cancer	*Vivo:* HT29 cancer cells xenograft male nude mice (5 or 10 mg/kg fucoidan: intraperitoneally injected every 2 days for 30 days)	↓ Angiogenesis ↓ VEGF and CD31	[27]
Fucoidan (from *Fucus vesiculosus*) (Sigma Aldrich)	Murine Lewis lung carcinoma	*Vitro:* Murine Lewis lung adenocarcinoma cell line (50–400 µg/mL for 24 h) *Vivo:* Lewis lung carcinoma xenograft male C57BL/6 mice (1 or 3 mg/day by intragastric gavage 7 days prior to tumor inoculation)	↓ Migration ↓ MMPs ↓ Angiogenesis and metastasis ↓ NF-κB ↓ VEGF ↓ MMPs	[28]
Crude Fucoidan (from *Fucus vesiculosus*) (Sigma Aldrich)	Murine breast cancer	*Vitro:* 4T1 cancer cells (50–200 µg/mL for 48 h) *Vivo:* 4T1 cancer cells xenograft BALB/c mice (5 or 10 mg/kg b.w. fucoidan intraperitoneally injected every 2 days for 20 days)	↓ VEGF in vitro and in vivo ↓ Tube formation in vivo ↓ Angiogenesis in vivo ↓ Lung metastases	[29]
Fucoidan (source not specified) (Sigma Aldrich)	Human breast cancer	*Vitro:* MDA-MB-231 cell line (6.25–25 μg/mL for 48 h)	↓ Invasion and migration of MDA-MB-231 cells ↓ HIF-1α and HIF-1 target genes (TWIST, Snail, CAIX and GLUT-1) in hypoxic conditions ↓ N-cadherin and vimentin and ↑ E-cadherin and ZO-1 ↓ EMT	[30]
Fucoidan (source not specified) (Sigma Aldrich)	Human prostate cancer	*Vitro:* DU-145 cell line (100–1000 μg/mL) *Vivo*: DU-145 cancer cells xenograft athymic nude mice (20 mg/kg b.w. of fucoidan for 28 days by oral gavage)	↓ Angiogenesis and cell migration ↓ Hemoglobin content ↓ mRNA expression of CD31 and CD105 in tumor tissue ↓ JAK and STAT3 pathway ↓ Tube formation	[31]
Fucoidan (from *Laminaria japonica*)	Human triple-negative breast cancer	*Vitro*: MDA-MB-231 and HCC1806 cell line (125–2000 µg/mL fucoidan for 24/48 h) HUVECs (100–500 µg/mL for 18 h or incubated with conditioned medium from fucoidan-pretreated MDA-MB-231 and HCC1806 cells for 24 h) *Vivo*: Embryos of transgenic zebrafish Tg(fli1:EGFP) (0.1–2 mg/mL fucoidan for 48 h) or zebrafish xenograft model injected with fucoidan pre-treated GFP-expressing MDA-MB-231	↓ VEGFA, IGF-I, bFGF, MMP-2, and MMP-9 ↓ Migration and invasion in HUVEC and cancer cells ↓ Angiogenesis and metastatic capability in vivo	[32]
Fucoidan (from *Sargassum hemiphyllum*) (Hi-Q Marine Biotech International Ltd., Taipei, China)	Human hepatocellular carcinoma	*Vitro:* SK-Hep1 and HepG2 cell lines (200 µg/mL for 24 or 48 h)	↓ TGF-signaling Regulation of miR-29b-DNMT3B-MTSS1 axis ↓ Invasion and metastasis ↓ EMT (↓ N-cadherin; ↑ E-cadherin) ↓ ECM degradation (↓ MMPs; ↑ TIMP)	[33]
Low molecular weight flucoidan (LMWF) 760 Da (from *Sargassum hemiphyllum*) (Hi-Q Marine Biotech International Ltd.)	Human bladder cancer cells	*Vitro*: T24 cell line (25–100 µg/mL) HUVECs (25–100 µg/mL) *Vivo:* T24 cancer cells xenograft BALB/c nude mice (80, 160 or 300 mg/kg b.w. per day LMWF orally administered for 30 days)	↓ VEGF and HIF-1α ↓ PI3K/AKT/mTOR/p70S6K/4EBP-1 ↓ Migration and invasion of T24 cells in hypoxic conditions ↓ Tube formation in HUVECs in hypoxic conditions ↓ Tumor angiogenesis in vivo	[34]
Fucoidan (from *Sargassum fusiforme*)	Human hepatocellular carcinoma	*Vitro:* SMMC-7721, Huh7 and HCCLM3 cell lines (500–30,000 µg/mL for 24/48 h) *Vivo:* HCCMLM3 xenograft male BALB/c nude mice (1 g/kg b.w. orally administered fucoidan for 21 days)	↓ Migration and invasion ↓ Invadopodia-related proteins (Src, Cortactin, N-WASP, ARP3, CDC42, MMP2, MT1-MMP) ↓ Integrin αVβ3 ↓ Tumor growth and lung metastatic foci in vivo	[35]
Fucoidan FP08S2 (from *Sargassum fusiforme*)	Human lung cancer	*Vitro:* HMEC-1 cells (4.21, 8.42, 16.84 μM for 8, 12 or 24 h) [36] (25–800 µg/mL for 72 h) [37] *Vivo:* CAM (2 days after 50–150 μg/egg fucoidan injection) A549 cancer cells xenograft nude mice (0.5 or 10 mg/kg b.w. via tail vein injection every day for 27 days)	Block of VEGFR2/Erk/VEGF signaling pathway in HMEC-1 cells ↓ Migration and invasion of HMEC-1 cells ↓ Tube formation and VEGF and HIF-1α ↓ Tumor growth and microvessel formation in vivo	[36,37]
Fucoidan (from *Turbinaria conoides*):	Human pancreatic cancer	*Vitro:* MiaPaCa-2 and Panc-1 cell line (25–100 µg/mL for 48 h) HAEC cells (12.5–100 µg/mL for 3 h) *Vivo:* CAM (4 days after 250–1000 µg/mL fucoidan injection)	↓ Migration in MiaPaCa-1 cells ↓ Tube formation ↓ Tubule junctions and neovascularization	[38]
Fucoidan (from *Undaria pinnatifida sporophylls*) (Dalian Aquaculture Group Co., Ltd., Dalian, China)	Murine hepatocarcinoma	*Vitro:* Hca-F cell line (100–400 µg/mL for 24 h) Murine LEC (50–200 μg/mL for 48 h in a hypoxic environment) [39] Human LEC (100–400 μg/mL for 24, 48 or 72 h) [40] *Vivo:* Hca-F cancer cells xenograft male 615 mice (15 mg/kg and 30 mg/kg b.w. abdominally injected once a day for 3 weeks) [39] or (30, 60, 120 mg/kg intragastrically administered for 14 days) [40]	In hypoxic conditions: ↓ HIF-1α/VEGFC and HGF ↓ PI3K/Akt/mTOR pathway in murine LEC ↓ NF-κB/PI3K/Akt pathway in human LEC ↓ MMP-2, 9; ↑ TIMP ↓ Lymphangiogenesis; ↓ PROX1 and VEGF3 ↓ Metastasis and lymphangiogenesis ↓ HIF-1α and VEGF-C ↓ VEGF3 and lymphatic vessel density	[39,40]
Fucoidan (source not specified)	Human lung cancer	*Vitro*: A549 and H1650 NSCLC cell lines (10,000 or 16,000 µg/mL for 48 h) *Vivo*: NSCLC cancer cells xenograft model (25 mg/kg b.w. via oral gavage every day for 14 days)	↓ Angiogenesis via m-TOR pathway ↓ Migration and invasion via EMT ↓ VEGFA, N-cadherin; ↑ E-cadherin	[41]
Oligo-fucoidan (source not specified)	Human hepatocellular carcinoma	*Vivo*: Transgenic human hepatocellular carcinoma zebrafish model (fucoidan 6.6 mg/kg b.w. oral gavage in combination with WNK463, Regonafenib or Rafoxanide twice a week for one month)	↓ WNK1–OSR1–PPP2R1A axis ↓ Migration	[42]
Fucoidan-coated manganese dioxide nanoparticles (Fuco-MnO_2_-NPs)	Human pancreatic cancer	*Vitro*: AsPC-1 and BxPC-3 cell lines (0.5–20 µg/mL for 48 h) *Vivo*: BxPC3 cancer cells xenograft mice (NPs 200 ng/50 µL and fucoidan 100 ng/50 µL by intratumoral injection weekly up to 30 days)	Suppression HIF-1α in hypoxic conditions ↓ VEGFR2 and CD31	[43]

↑: increase; ↓: decrease; **4EBP-1**: eukaryotic translation initiation factor 4E-binding protein 1; **Akt**: protein kinase B; **ARP3**: Actin Related Protein 3; **bFGF**: fibroblast growth factors; **b.w.**: body weight; **CAIX**: carbonic anhydrase IX; **CAM**: chorioallantoic membrane; **CD31**: cluster of differentiation 31; **CDC42**: cell division control protein 42 homolog; **CXCR4**: alpha-chemokine receptor specific for stromal-derived-factor-1; **Da**: Dalton; **DNMT3B**: DNA methyltransferase 3 beta; **ECM**: extracellular matrix; **EGFP**: enhanced green fluorescent protein; **EMT**: epithelial mesenchymal transition; **ERK**: extracellular signal-regulated kinase; **FLT**: fms related tyrosine kinase; **GLUT-1**: glucose transporter protein-1; **KDR**: kinase insert domain receptor; **KDRL**: KDR-like; **HAEC**: human aortic endothelial cells; **HIF-1α**: hypoxia-inducible factor 1α; **HGF**: hepatocyte growth factor; **HMEC-1**: human microvascular endothelial cells; **HUVECs**: human umbilical vein endothelial cells; **IGF-I**: insulin-like growth factor-I; **mLEC**: mouse lymphatic endothelial cells; **MT1-MMP**: membrane-type 1 matrix metalloproteinase; **MMPs**: metalloproteinases; **MSCs**: human mesenchymal stem cells; **mTOR**: mammalian target of rapamycin; **MTSS1**: Metastasis suppressor protein 1; **NF-κB**: nuclear factor kappa-light-chain-enhancer of activated B cells; **NOD/SCID**: immunodeficiency/non-obese diabetic mice; **N-WASP**: Neuronal Wiskott-Aldrich Syndrome Protein; **OEC**: human blood derived outgrowth endothelial cells; **p70S6K**: phosphoprotein 70 ribosomal protein S6 kinase; **PI3K**: phosphatidylinositol-3 kinase; **PDGF**: platelet-derived growth factor; **p-AKT**: phosphorylated protein kinase B; **p-ERK1/2**: phosphorylated extracellular signal-regulated kinases ½; **PPP2R1A**: protein phosphatase 2A (pp2a) subunit ppp2r1bb; **PROX1**: prospero homeobox protein 1; **Rafoxanide**: OSR1 (oxidative stress responsive 1) inhibitor; **SDF-1**: stromal derived factor-1; **src:** steroid receptor coactivator; **TIMP**: tissue inhibitor of metalloproteinase-1; **WNK463**: WNK1 (lysine-deficient protein kinase-1) inhibitor; **VEGF**: vascular endothelial growth factor; **VEGFR2**: vascular endothelial growth factor receptor 2; **ZO-1**: zonula occludens-1.

## Data Availability

Data available in publicly accessible repositories (PubMed, clinicalTrials.gov).
